# Using Aboriginal participatory action research within a mixed methods study exploring COVID-19 vaccination uptake in Aboriginal women: a methodological framework for co-design

**DOI:** 10.3389/fgwh.2026.1781179

**Published:** 2026-07-29

**Authors:** Anne-Marie Eades, Tilsa Guima Chinen, Alison Walton-Blane, Eliza Razak, Ananda Buckley, Katie Attwell, Zoe Bradfield, Juli Coffin, Sandra Eades, Martyn Symons, Hueiming Liu, Margaret O'Connell, Francine Eades, Melanie Robinson, Mima Bull, Jodie Ingrey, Jenna Greaves, Talila Milroy, Jo-Anne Morgan, Rachel Foong, Jasmine Helwig, Lesley Nelson, Tiana Culbong, Samantha J. Carlson, Christopher Blyth

**Affiliations:** 1 Curtin University, Perth, WA, Australia; 2 The Kids Research Institute Australia, Perth, WA, Australia; 3 WA Department of Health, Perth, WA, Australia; 4 University of Western Australia, Perth, WA, Australia; 5 Murdoch University, Perth, WA, Australia; 6 University of Melbourne, Melbourne, NSW, Australia; 7 The George Institute for Global Health, Newtown, NSW, Australia; 8 WA Department of Health, East Metropolitan Aboriginal Health, Perth, WA, Australia; 9 WA Department of Health, Service Child and Adolescent Health Services, Perth, WA, Australia; 10 South West Aboriginal Medical Service, Bunbury, WA, Australia; 11 Babbingur Mia, South Coastal Health & Community Services, Rockingham, WA, Australia; 12 WA Department of Health, Communicable Disease Control Directorate, Perth, WA, Australia

**Keywords:** Aboriginal, APAR, co-design, communicable disease, pregnancy, vaccine hesitancy, yarning

## Abstract

The COVID-19 pandemic evidenced the critical role of vaccination in reducing the severity of illness and the risk of infection. Despite the policies and efforts of governments to increase vaccination uptake, COVID-19 immunization among Australian Aboriginal pregnant women is lower compared to vaccine uptake of non-Indigenous pregnant women. To date, there have been no national Aboriginal Participatory Action Research (APAR) co-designed Aboriginal-led studies exploring the factors influencing uptake of COVID-19 vaccination in Aboriginal women of childbearing age. This Aboriginal-led research was conducted with Aboriginal women of childbearing age and non-Aboriginal mothers of Aboriginal children in the Great Southern region of Western Australia (WA). The study aimed to explore the factors influencing COVID-19 vaccine knowledge, confidence, and uptake and was conducted in consultation with Aboriginal Elders and health service partners in the region. This research employed an explanatory sequential mixed methods design, with co-design principles embedded at every stage of the study. Using Western quantitative methods and Indigenous qualitative methodologies, the research completed 110 surveys and conducted 5 Yarning Circles. Participants reported safety and trust as key factors influencing vaccination decision making. Although the vaccine mandate increased vaccine uptake, the study found a decline in vaccination following the end of the mandate. Participants also reported ongoing challenges following the COVID-19 pandemic as lack of engagement and access to health information and services. The findings underscore the importance of cultural legitimacy in research and the need for sustained partnerships between Indigenous communities and health researchers to foster trust and promote health equity. This paper outlines the methodological framework implemented in the Ngarngk Koolangka Moorditj Yarning Project (NKMY) conducted in Australia (2022–2024).

## 1 Introduction

Western research methodologies historically have been influenced by unethical and harmful research practices, which caused detrimental effects to First Nations peoples (1, 2). Indigenous research methodologies contest the notion of scientific knowledge production and its detrimental effect on First Nations peoples by denouncing and deconstructing Western methodologies and its ongoing colonial impact (2, 3). In doing so, Indigenous research methodologies position and validate Indigenous knowledge systems and lived experiences as core and foundational to academic knowledge production (2). In this context, Aboriginal Participatory Action Research (APAR) emerges as an Indigenous methodology grounded in cultural legitimacy, Community empowerment, and ethical fidelity, deeply involving Community members throughout the research process (4). This ensures that cultural norms and values are not only respected but also intricately woven into the research fabric, thereby addressing the longstanding issues of power imbalance, mistrust, and misrepresentation that often plague conventional research methods (2). This method also fosters genuine partnerships based on trust and mutual respect, yielding richer and more nuanced data and insights (5). Furthermore, APAR's action-orientated nature ensures that research is Community-driven, implementing practical solutions that resonate with the strengths, needs, and aspirations of Aboriginal communities. By advocating for reciprocity, respect, and responsibility, APAR guarantees that the research serves the best interests of Aboriginal communities, safeguards the rights of Communities to control their data, and enhances their well-being, making it an ethically sound and impactful approach for research conducted within Aboriginal contexts (6).

The self-determination principles and collaborative partnerships grounding APAR and Indigenous research methodologies require dynamic frameworks and dialogical processes that challenge power imbalance in conventional Western research models (7). Research that uses a co-design approach is considered best practice within APAR, as it prioritizes Aboriginal voices and expertise, and provides opportunity for empowerment (1, 8). Applied in the context of healthcare, co-design is intended as a tool for facilitating cultural responsiveness, creating culturally safe healthcare systems, and empowering Community-driven polices to improve healthcare access and delivery (9). Although co-design is considered the optimal approach within Aboriginal research, there is limited consensus or guidance for what constitutes effective co-design (10, 11). Therefore, it is perhaps unsurprising that the interpretation and implementation of co-design is subject to variation. Anderson et al. 2022 (10) outlined a set of six key principles associated with best practice for co-design in health with First Nations Australians: First Nations leadership, culturally grounded approach, Respect, Benefit to First Nations Communities, Inclusive partnerships, and Transparency and Evaluation. Whilst these principles provide a strong thematic and ethical framework for using co-design in First Nations communities, a methodological framework outlining how optimal co-design principles are applied is required. This paper outlines a framework for co-design used within the Ngarngk Koolangka Moorditj Yarning (Mother and Baby Solid Yarning) Project (NKMY) which explored experiences and perceptions of the COVID-19 vaccination uptake in childbearing women in Aboriginal communities in Southwest Western Australia.

### 1.1 Project rationale

There are significant global inequities in access to vaccinations for Aboriginal people (12). In Australia, vaccination uptake since the COVID-19 pandemic was generally lower within the Aboriginal and Torres Strait Islander (hereafter respectfully termed Aboriginal) Communities (with gaps ranging from 7% to 31% depending on dosage) when compared to non-Aboriginal people (13, 14). The reasons for this are multifactorial and include a combination of mistrust in governments and healthcare providers from colonization-induced trauma, fears about health vulnerability, and doubts about vaccine safety (15–18). More recently, the COVID-19 pandemic evidenced the critical role of vaccination in reducing the risk of harmful consequences related to contracting a severe COVID-19 infection and limiting virus transmission (19). As the evidence shows, if infected, pregnant women are more likely to develop adverse outcomes, are at higher risk of pre-term birth, and their infants are at increased risk of requiring neonatal intensive care (20, 21). Therefore, maternal vaccination is key to ensuring the health and wellbeing of mothers and their infants.

Although COVID-19 vaccine hesitancy is reportedly higher in pregnant mothers than in non-pregnant women of the same age (22, 23) recent studies reveal the recommended maternal vaccine coverage rate was 20% lower in Aboriginal pregnant women compared to non-Aboriginal pregnant women (24). Studies report that Aboriginality, as well as age, and gender, are among the socioeconomic factors associated with lower levels of confidence in COVID-19 vaccine safety, vaccine willingness, and uptake (25, 26). For Aboriginal women, this hesitancy could be compounded by broader Community concerns about COVID-19 and vaccinations (22, 27, 28).

As evidenced from studies of non-Indigenous Australian women on COVID-19 vaccination, vaccine uptake is influenced by a number of factors including lack of awareness of the potential risks of being pregnant and unvaccinated, associations with serious illness from COVID-19, fear of side effects for both mother and baby, insufficient information about COVID-19 vaccinations, lack of information about COVID-19 vaccinations and lack of communication from health personnel (23, 29). These factors highlight the importance of improving confidence and trust in vaccine efficacy and safety in pregnant and childbearing women for public co-operation and vaccination uptake (25, 26).

Besides addressing these factors, specific Community-led and evidence-based approaches are required to facilitate uptake in Aboriginal women (30). During the COVID-19 pandemic, Aboriginal people led their own COVID-19 coping and vaccination strategies (31). This response aimed to prevent high rates of mortality and morbidity from the previous H1N1 epidemic in 2009 and the health and social gap impacting healthcare access for rural and remote communities (31, 32). With a focus on keeping Elders and the Community safe, First Nations health professionals and Aboriginal Community Controlled Health Organization's (ACCHO) united within their Communities to advocate for testing and vaccination rollouts and for the repurposing of existing resources. They also supported and engaged members of the Community on COVID-19-related issues and closed their borders to protect vulnerable people within the Community (33, 34). By 2021, 148 cases of COVID-19 in First Nations people in Australia had been reported, including 15 hospital admissions, one person in intensive care, and no deaths (31). These numbers contrast with the 362,677 cases of COVID-19, including 2,226 deaths, reported in Australia by the end of December 2021 (35). The success of these strategies relies on the Community's self-determination approach, on their ability to design and implement health responses through effective partnerships with government and non-government services, and on their power to act accordingly (31, 33).

However, two years after the end of the pandemic, there remained limited resources available to support Aboriginal women to make decisions regarding the COVID-19 vaccines. The Ngarngk Koolangka Moorditj Yarning (NKMY) study explored the experiences and factors influencing COVID-19 vaccine decision-making in Aboriginal women of child-bearing age in the Great Southern region in Western Australia. The study also sought to develop Culturally appropriate recommendations and resources to increase vaccine confidence and uptake.

## 2 Methods

**Figure 1:**
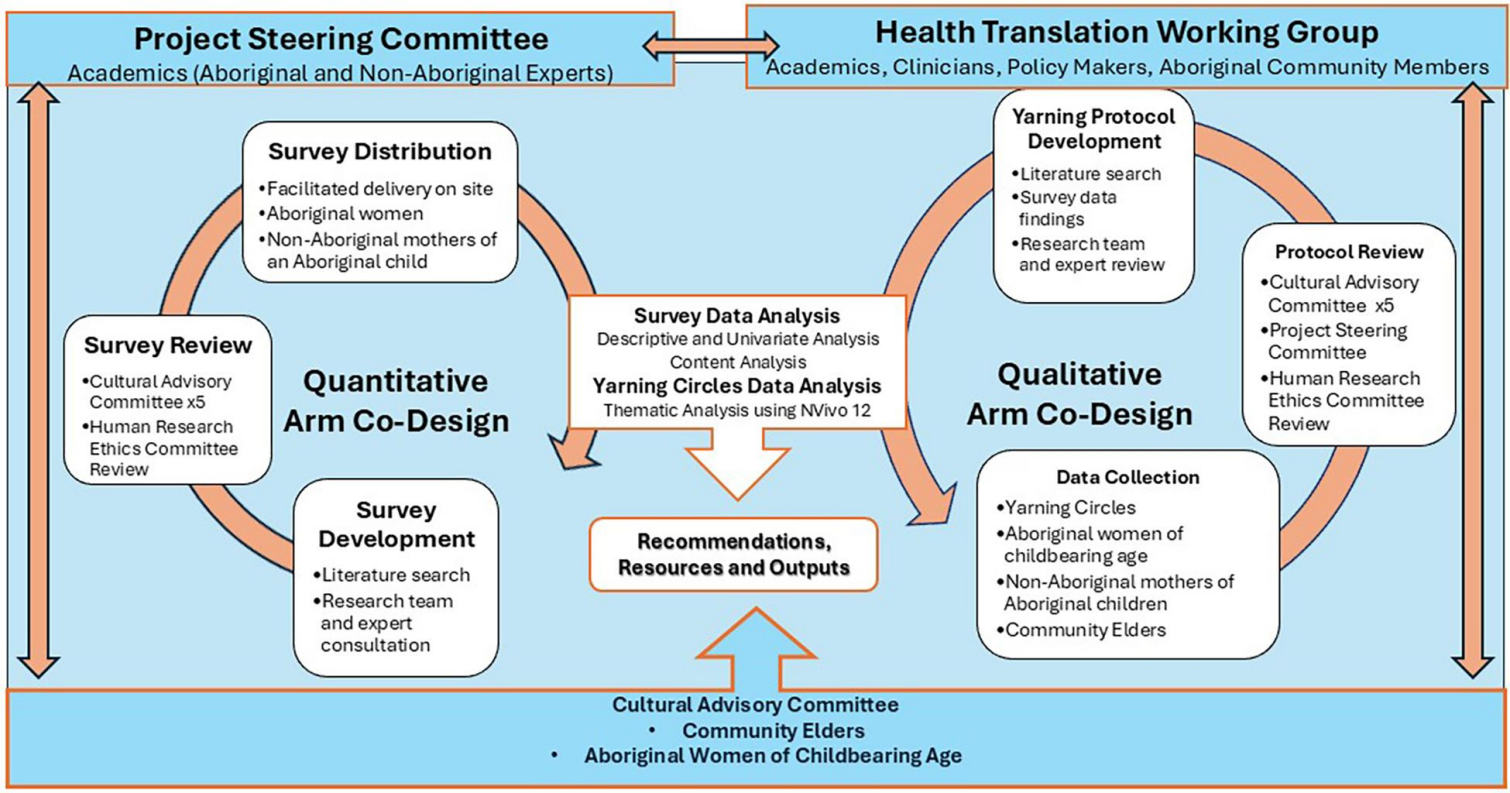
The Ngarngk Koolangka Moorditj Yarning co-design framework.

The APAR process supports a forum for combining postcolonial and hybrid knowledge in ways that inform interventions, theories, and advocacy (36). Combining Western and Indigenous expertise and knowledges has the potential to allow clinical and research teams to work at the intersection of Indigenous and Western academic knowledge and practices (37, 38). This research employed an explanatory sequential mixed methods approach, with a quantitative survey informing Yarning Circles (Figure 1). Yarning Circles were used, as they create relationships and provide a safe space for Aboriginal people to share their experiences and build new knowledge through storytelling (39, 40). As an established Indigenous research methodology, Yarning reveals rich, insightful, and valuable contexts, as well as allowing for the transfer of intergenerational knowledge sharing that may not be identified in traditional forms of Western methodology (3, 39, 40). It is a fluid process of knowledge sharing and respectful communication that is grounded in cultural connections, providing a culturally safe space for honest and open conversations (40). More than merely a data collection tool, Yarning fosters a relational process that builds essential trust and rapport, particularly important in contexts marred by historical exploitation and mistrust. The use of Yarning in the study aligns with ethical research practices, promoting reciprocity, respect, and ensuring beneficial outcomes for participants, contributing to Community capacity building by valuing the participants voices (39).

The mixed methods approach was underpinned by an APAR framework and utilizes cyclical, dynamic, and reflective processes that enable culturally safe and Community-driven solutions (41, 42). The methodology implemented in the project additionally incorporates a co-design process across all stages of the research. The combination of a quantitative survey and Yarning Circles informs the data collection process, with ongoing oversight from the Cultural Advisory Committee (CAC), the Project Steering Committee (PSC), and the Health Translation Working Committee (HTWC). The research translation strategies and the project outputs were also co-designed and led by the CAC.

### 2.1 Researchers' positioning

The study was conducted by an interdisciplinary and intercultural team led by senior Noongar (Minang) researcher AE with over twenty years of experience working in Aboriginal Health, qualitative research, and health service research. The project was supported by two Aboriginal researchers, MB Wiradjuri and Aboriginal Health Practitioner, and JG Yamatji Naaguja Nyarlu and Clinical Nurse Manager. The research team was comprised of two post-doctoral non-Indigenous researchers, AWB and TG, and two non-Indigenous research assistants, ER and AB. The combination of interdisciplinary background of the team and their expertise in quantitative, mixed-methods, and co-designing focused on human factors, epidemiology, and Aboriginal public health contributed to the development of the study. Both Aboriginal (AE, JC, SE, FE, MR, MB, JG, TM, JH, LN, TC) and non-Aboriginal (TGC, AWB, ER, AB, ZB, MS, HL, MO, JI, JM, RF, SC, CB) Chief Investigators also provided guidance and mentoring for the data analysis, ensuring an accurate interpretation of the participants' views and experiences shared in the research.

The framework seen in Figure 1 implements engagement protocols and establishes the co-design processes for research, positioning Aboriginal leadership at the forefront with Community voices and the ACCHO sector. In the NKMY project, the framework established communication processes for shared decision making through the projects Governance bodies. In doing so, the framework allowed for an effective and appropriate research design to identify and discuss the factors influencing vaccine decision-making for Aboriginal women of childbearing age and non-Aboriginal mothers of Aboriginal children under the guidance of Community Elders.

### 2.2 Study governance

Consistent with APAR principles, the project was overseen by the following Indigenous governing structures:

#### 2.2.1 Project cultural advisory committee

The Cultural Advisory Committee (CAC) was comprised of local Aboriginal Elders, Aboriginal women of child-bearing age, senior Aboriginal women, and local Community members. A CAC was established within geographical target areas within each research site. The CAC recommended the Noongar name for the project: Ngarngk Koolangka Moorditj Yarning (NKMY), which translates to “Mother and Baby Solid Yarning”, and was involved throughout the different stages of the research, adding reliability and trustworthiness of the interpretation and findings. The addition of the CAC in the Governance body contributes to decolonizing research practice by ensuring that Indigenous knowledges, and principles of self-determination and sovereignty are upheld in the research process (3).

#### 2.2.2 Project steering committee

The Project Steering Committee (PSC) was comprised of Aboriginal and non-Indigenous Chief Investigators, Partner Investigators, Associate Investigators, and service partners. The project was led and governed by senior Aboriginal researchers with expertise in Aboriginal health, co-design, APAR, and quantitative and qualitative research methods. Non-Indigenous researchers included health practice leaders and clinical experts who are leading COVID-19 vaccination research, and advisers on health policy. This diverse research team formed the PSC within the research and met bi-monthly to provide input on the development and implementation of high-quality collaborative multi-disciplinary research.

#### 2.2.3 Health translation working committee

A Health Translation Working Committee (HTWC) of experts provided planning and implementation of expert knowledge for research translation related to academic and policy outputs. The HTWC included Investigators, Aboriginal Consumer Reference Committee, and key stakeholders. At quarterly meetings, the HTWC members were given project updates and asked to inform targeted recommendations regarding service delivery, information, education and communication materials, policy briefs and manuscripts with the Community.

### 2.3 Participants recruitment

The research was conducted in partnership with two Aboriginal health services (also known as partner organizations), one in Metropolitan Perth and the other in the Southwest region of Western Australia. The metropolitan service is an Aboriginal health service (AHS) operating within a mainstream health service, and the regional service as an ACCHO. At both sites, Community Connectors supported the recruitment of research participants for the surveys and Yarning Circles. Participants were recruited through the partner organizations, community organizations, and community connectors, posters and flyers. A community-based convenience sampling approach was considered a more appropriate and culturally acceptable recruitment strategy for engaging participants across the research sites.

### 2.4 Quantitative study design, sample, and analysis

A comprehensive cross-sectional survey was co-designed with the Cultural Advisory Committee (CAC) representatives from each research site, including Bunbury (Eaton), Collie, Busselton, Katanning, and Rockingham. Participants were Aboriginal women aged 18 years and over who were residents of Western Australia (WA) and who were either pregnant or trying to conceive during the COVID-19 pandemic. Following consultation with the Cultural Advisory Committee (CAC), Non-Aboriginal women who were pregnant during the COVID-19 pandemic, as well as Non-Aboriginal mothers of an Aboriginal child, were also included in the study.

Researchers and Community Connectors engaged in and facilitated delivery of the survey to partner organizations. Participants were provided with a mobile tablet with access to the internet and given the opportunity to ask for assistance with completing the survey if needed. The survey was also distributed in the local Community using posters and flyers which were also displayed in the partner organizations. Following Jones, Gilroy (43), a safety protocol was incorporated into the survey. This included a debriefing form that could be administered in person, where the survey was undertaken using facilitated delivery or administered online and with the opportunity for researchers to follow up with participants at their request.

The cross-sectional survey consisted of quantitative data (e.g., categorical and Likert-scale type questions) with complementary short-answer data. Questions related to demographics, pregnancy status, levels of confidence in the COVID-19 vaccine, experiences of vaccine uptake, COVID-19 infections, concerns related to the COVID-19 vaccine, factors influencing the uptake of the COVID-19 vaccine, and intentions or behavior relating to COVID-19 vaccination. Baseline data for developing the quantitative survey was drawn from existing vaccine social science instruments, including the COVID-19 vaccine Knowledge, Attitude, Practices, and Concerns (KAPC) questionnaire (44) and the Vaccination Concerns in COVID-19 Scale (VACCS; 45). Both surveys have been trialed and validated in the general population prior to use in this study and served as a guiding tool in the development of the cross-sectional survey used in this study.

The drafted survey was distributed amongst the internal study team and PSC for initial feedback and amendments before being endorsed by the CAC. Feedback from these groups informed the content and wording of questions and responses in the final survey. The co-design process increased data trustworthiness and participant safety, as well as ensuring relevance to the Community. The survey was designed to take approximately 20 min to complete.

### 2.5 Sample size calculation

According to the Australian Bureau of statistics 2021 data, there were 5,919,731 [Supplementary Appendix (46)] women of childbearing age (age 15–49 years) in Australia. The total number of Western Australia (WA) births reported was 34,065 and the number of births to Aboriginal women in WA was reported as 2,895 (47). The WA government previously defined an individual as fully vaccinated against COVID-19 if they had received the primary course of two vaccinations, that is, double vaccination (48). As of March 2023, the proportion of double vaccination in WA for people aged over 16 years was 92%, however, the proportion of the vaccination uptake in the Aboriginal population of WA was lower at 85% (13). It is not known how many pregnant Aboriginal women chose to vaccinate. Assuming an 85% of COVID vaccine uptake, a 95% confidence interval and a margin of error of +/- 5%, a sample size of 184 was estimated. This corresponds to a standard error of 0.025 and a relative standard error of 3%.

### 2.6 Data collection

The survey was developed and distributed using the online survey tool Qualtrics (49). Development of the survey commenced in February 2023 and was informed by validated vaccination social science instruments, as mentioned in section 1.4: Quantitative study design, sample, and analysis. The draft survey was subject to review by the study team, PSC and CAC, keeping to a co-design process, implementing feedback informing the final survey content and wording. A condensed version of the final survey is available in the Supplementary Appendix 10.1 “Ngarngk Koolangka Moorditj Yarning Project Survey”.

Participants were recruited through Partner organizations and trained Community Connectors. Surveys were distributed electronically by Partner organizations and Community Connectors, adopting a snowball sampling method, where participants were encouraged to share information about the study to mothers of Aboriginal children within their community. The survey was completed on iPads provided to sites; responses were collected electronically through Qualtrics and were stored securely within password protected Microsoft Teams folders accessible only to authorized members of the research team. All study data collected was de-identified at the source to ensure participant confidentiality.

### 2.7 Data analysis

Quantitative data was analyzed using the IBM Statistical Package for Social Sciences (SPSS) version 28.0. Descriptive and univariate statistics, including measures of central tendency, frequency counts, and group-based comparisons were used and supplemented by multinomial logistic regression examining associations between demographic factors, health experiences, and vaccine beliefs with self-reported vaccination status during pregnancy and conception. Normality of data was assessed using the Kolmogorov–Smirnov test and the appropriate test selected based on the results. An alpha value of 0.05 was used to determine statistical significance and effect sizes, e.g., Cohen's D, correlation coefficients reported where applicable. Qualitative responses were categorized using content analysis and used to complement the quantitative findings. This allowed for identification of the key issues associated with the choice to vaccinate against COVID-19 in women of childbearing age.

### 2.8 Qualitative study design, sample, and analysis

Yarning is conversational in nature, requires meticulous planning, knowledge of cultural protocols, deep listening, and skilled facilitation (40). As an established Indigenous research methodology, the implementation of Yarning Circles aligned with the confines of this study (39, 40). Informed by the initial findings from the survey, the Yarning Circle questions focused mostly on the women's impressions and experiences. The survey findings informed the development of the Yarning Circle discussion and how it was guided, enabling further exploration of themes identified in the survey responses. Topics identified in the survey where further explored during the Yarning Circles, these included, attitudes influencing vaccine decision-making, perceptions of vaccine safety and effectiveness, sources of vaccine information, trust in healthcare providers and institutions, and experiences and views relating to the WA vaccine mandate.

During the sessions, participants engaged in courageous conversations revealing the impact that the COVID-19 mandates had on their communities and their experiences with the COVID-19 vaccine. Additionally, the Yarning Circles provided an opportunity for intergenerational knowledge as the Elders guided members of their family (daughters and grandchildren) participating in the study. Through their leadership and participation, the Elders guided the yarns and created a culturally safe space for the research.

By entrusting the research, Elders and Community stakeholders validated the integrity of the Yarning Circles. The collective fabric of the Yarning Circles conceives the method not as limited to a data collection tool but as embedded in reciprocity and therefore, as a vehicle for Community advocacy and social change. By framing the research according to cultural protocols and unpacking the perceptions and experiences of Aboriginal women of childbearing age, the Yarning Circles added a layer of in-depth analysis to the quantitative data complementing the initial survey findings.

The Yarning Circle questions were developed through a co-design process involving the CAC, PSC, partner organizations and governance committees. Draft questions informed by the survey findings were reviewed and refined based on stakeholder feedback. The final Yarning Circle questions were endorsed by the CAC. The endorsed questions were submitted for the final ethics approval. The CAC provided oversight of data collection activity and engagement with the Aboriginal community. The Yarning Circles were facilitated by the lead researcher and research team using approved Yarning Circle questions.

The conversations captured in the Yarning Circles exhibited the harmonizing of each voice, resonating across generations, from the young mothers to the grandmothers in the room. The space that was created in these Yarning Circles ensured that the courageous conversations were conducted in a culturally safe environment. This dynamic proposes a holistic approach to health policies as addressed in the recommendation section.

### 2.9 Participants and data collection

The mixed methods data collection approach allowed for the participant criteria to be broad in order to capture the views and experiences of women of child-bearing age. This approach also enabled the study to capture the experiences of women who had been pregnant or had babies during the COVID-19 pandemic. The two partner organizations contacted Aboriginal women through Community Connectors employed by the project. Flyers were designed and distributed in collaboration with the research team, partners, and the Community, along with the research investigators' existing networks. The Community Connectors recruited 51 participants through purposeful, convenience, homogenous, and snowball sampling to participate in 5 Yarning Circles across both sites. Participant numbers were estimated based on the number of Aboriginal women of child-bearing age living in the two regions who access AHSs. Aboriginal staff and Community Connectors from partner organizations collaborated with research team members to ensure that data collection activities and interactions with Aboriginal Community members were culturally relevant and secure. The Yarning Circles were conducted in May 2024 across the different research sites: Eaton (Monday 6th), Busselton (Tuesday 7th), Collie (Wednesday 8th), Katanning (Thursday 9th), and Rockingham (Tuesday 17th).

The number of Yarning Circle sessions continued until data saturation was reached. The guiding questions for the Yarning Circles were submitted to the Western Australian Aboriginal Health Ethics Committee for approval before starting the data collection. The questions expanded on the experiences of Aboriginal women of child-bearing age in relation to the survey findings. The Yarning Circles also explored participants' views on the information provided to them by government and local health providers, about COVID-19 illness and vaccinations. The sessions concluded with recommendations for the development of culturally secure resources and relevant information for the Community. Participants were provided with an opportunity to have an individual interview if preferred, rather than participating in the Yarning Circle; however, these were not requested. At the end of the Yarning Circle sessions, participants were provided with gift cards in appreciation for their time and knowledge.

### 2.10 Data analysis

Qualitative analysis, data sorting, and coding were conducted using NVivo software. A coding framework was developed, partly from *a priori* theoretical perspective and partly from the main themes established through close reading and thematic analysis of transcripts. Multiple research team members were involved in developing the coding framework to avoid idiosyncratic interpretation. Analysis was thematic, by application of this coding framework to the interview transcripts and notes. The cyclical nature of the APAR process included confirming with participants the interpretations and findings of qualitative research to ensure accuracy of the views are represented. The CAC guided the direction of the data analysis through discussion, Community consultation, and feedback. Reporting data followed the Consolidated Criteria for Reporting Qualitative Research (COREQ) checklist (50). The findings from the Yarning Circles were presented to the CAC, the project's key stakeholders, and service providers.

### 2.11 Data crystallization

The survey results informed the development of the qualitative topics for discussion, and the findings from the survey were further explored in the Yarning Circles. Further triangulation of qualitative and quantitative data was conducted over the course of the research by the research team working closely with Chief Investigators, with feedback from the Governance Committees. The findings were expected to provide information and guidance for future interventions or changes in service delivery for Aboriginal women of child-bearing age, as well as methodological recommendations for information dissemination and improving vaccine acceptability for these Aboriginal women.

### 2.12 Ethics

This research received ethics approval from the Western Australian Aboriginal Health Ethics Committee # 1205 and the University of Technology Sydney Human Research Ethics Committee # ETH23-7990. Ethics amendments were submitted as required for the duration of the research.

The research was conducted in accordance with the NHMRC National Statement on Ethical Conduct in Human Research and Guidelines for Ethical Conduct in Aboriginal Health Research (51). All data records are managed according to National Health and Medical Research Council (NHMRC) ethical guidelines and University of Technology Sydney (UTS) standards and protocols. The research honored the rights of Aboriginal peoples to have control over their Communities, resources, and Country, and in the creation, collection, access, analysis, interpretation, management, dissemination, and reuse of data (52).

Full informed consent was obtained from all study participants. Participants were provided with an information statement and consent form and were given the opportunity to ask questions about the research before providing written consent. In the survey, the consent form was electronic and contact details for the research team were provided. Participation was voluntary, and participants were informed that they could withdraw from the research at any point without negative consequences to their participation in any program or service. Participants in the Yarning Circles could also ask to have information they have disclosed to be withheld from the research. As the survey was anonymous at source, it was not possible to remove participant data once the survey was started. Participants were made aware of this. Participants were given an assurance of confidentiality in all publicly available information and peer-reviewed publications. They were advised that data and identifying information would be deidentified or coded as soon as possible, and only decoded data would be stored on password protected computers and files that would only be accessible by members of the research team. All Yarning Circle and survey participants were renumerated for their time. Participants in the survey could collect their remuneration from the partner sites or by contacting the research team.

Audio recordings were transferred for transcription via a share-file held on the Universities' secure drive. All data records, including audio recordings and transcripts, consent forms, and survey data were managed according to ethical requirements. Consent forms are stored separately from any data collection forms. All hard copy and electronic data will be archived at UTS for a minimum of 7 years after the date of publication or research program completion, whichever is the latter in accordance with the requirements of the ethics bodies.

## 3 Results

**Figure 2:**
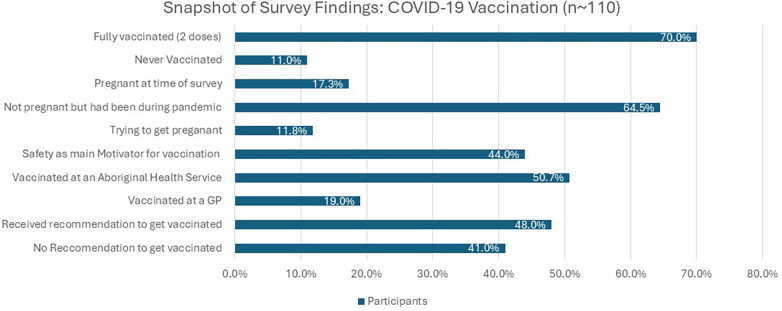
Snapshot of survey findings.

The table seen in Figure 2 provides an overview of the survey results. A total of 110 women responded to the survey, with 64.5% of participants being pregnant during the COVID-19 pandemic. Of the 110 respondents, 70% were fully vaccinated while 11% reported never receiving the vaccine. Among the vaccinated participants, safety was reported as the main motivator, with 50% receiving the vaccine at an AHS in contrast to 19% vaccinated at a GP. The survey findings revealed recommendations to get vaccinated were not widely received in the Communities, with 48% of participants receiving this recommendation compared to 41% who did not. These variations, including partial responses and open-ended comments, contributed valuable insight that supported the qualitative analysis.

Table 1 explores the themes identified through both the survey and Yarning Circles results.

**Table 1:** Survey and Yarning circles results.

Theme	Survey Data (Quantitative)	Yarning Circles Data (Qualitative)
Additional support during pregnancy	42 participants preferred face-to-face appointments 39 participants sought more written and/or in person information by health care professional 26 participants asked for reminders for vaccination appointments	
Unvaccinated participants	12 Participants never received a COVID-19 vaccine “*I feel like it shouldn't have been pushed on people. I also have OCD fear of contamination.”* (Aboriginal participant, survey - comment section) “*I don't believe it works.”* (Torres Strait Islander participant, survey—comment section). “*the unknown side effects and long-term health issues later on”* (Aboriginal participant, survey)	Unvaccinated participants experienced milder symptoms or avoided COVID-19, though some contracted COVID multiple times. “I didn't have vaccine. Well, I got COVID, but I wasn't bad at all.” (Aboriginal participant, Yarning Circle).
Vaccine hesitancy	*“Did not want to have vaccine while pregnant as there wasn't enough information”* (Aboriginal participant, survey—comment section)	Concerns: side effects, safety in pregnancy, effects on fertility Mistrust of experts & unclear health information Social media became a trusted source of information due to news overload
Mandate impact (WA 2021-22)	48% (52 participants) said mandates strongly influenced uptake 52% (57 participants) did not intend to receive further doses beyond the mandate “*I got the first two vaccinations. My children (who were) eligible got two vaccinations. If we are required to get more I will, but if it's not compulsory I won't be getting anymore unless it's necessary.”* (non-Aboriginal mother participant, survey—comment section). “*I might get more vaccine[s] now that it's not forced upon [us].”* (Aboriginal participant, survey—comment section).	Many participants expressed that COVID-19 mandates caused disempowerment due to forced vaccination requirements for accessing childcare centres, the risk of job loss, and a lack of personal choice. “*It creates more anxiety, worry, and fear. You're damned if you do and you're damned if you don't”* (Aboriginal participant, Yarning Circle) “*My son, he just got out of school and started working, and he felt threatened (and afraid) to lose his job”* (Aboriginal participant, Yarning Circle). “*I never had that vaccine. I made a choice and even quit my job when it all did come down because, as a Christian, I thought I'm not (going to) inject myself with something I knew nothing about.”* (Aboriginal participant, Yarning Circle). “*I still didn't because I didn't really want them to have that control over me.”* (Aboriginal participant, Yarning Circle)
Post-pandemic challenges	*“Did not want to have the vaccine while pregnant as there wasn't enough information,”* (Aboriginal participant, survey—comment section). “*It was uncertain at the time the effect*s on [their] unborn baby,” (Aboriginal participant, survey—comment section).	Many participants raised concerns over the information gap regarding trials and follow-up for vaccinated children. The women also reported confusion regarding COVID-19 status updates, post-pandemic measures after contracting COVID-19 and recommendations on annual COVID-19 shots like flu jabs. Other important concerns included post-vaccination fertility and menstrual health in young Aboriginal girls. “*Is there a trial for children? Has there been children already vaccinated? And has there been like checkups on the children since being vaccinated?”* (Aboriginal participant, Yarning Circle) “*I remember that was one of the reasons I was hesitant to get it. Because I wanted to have kids (was concerned that I could have infertility issues), (I wanted to know) does it affect me?”* (Aboriginal participant, Yarning Circle)

### 3.1 Vaccine hesitancy

During the sessions, concerns about vaccine side effects, particularly during pregnancy and on fertility, were frequently reported. Risks for pregnancy and breastfeeding, lack of clear information on the different types of vaccines and the vaccination process also ranked higher. Women reported worry about possible complications during pregnancy for their babies and considered that the information provided was not easy to understand. These risk perceptions during pregnancy and potential side effects on fertility were the main factors influencing vaccine hesitancy.

### 3.2 Mistrust

In response to the pandemic, the WA government delivered a COVID-19 vaccination program set to target 80% of the population. This campaign required an apparatus able to articulate the resources already existent with new and temporary resources like COVID-19 testing centers and vaccination clinics. In this context, information played a key component in connecting the public with these initiatives. However, the participants experienced mistrust, fear, and skepticism surrounding the development and dissemination of the vaccine. This was further exacerbated by unclear communication from health professionals and negative coverage from mainstream media. As a result, participants in the Yarning Circles reported feelings of news overload and an increased use of social media as a trusted source of information.

### 3.3 Mandate impact

WA implemented COVID-19 mandatory workplace vaccinations from 20 October 2021 to 10 June 2022 for workers in industries with high transmission risk and workforce critical to ongoing delivery of businesses and the function of the Community (53–55). WA's COVID-19 vaccine mandate influenced almost half of the participants to get vaccinated, primarily due to job security concerns. A total of 48% (52 participants) of the survey participants reported that the mandate strongly influenced their decision to receive a vaccine. Whilst the mandate motivated a significant number of participants to get vaccinated, the policy was problematic for many in the Community, as explored in the Yarning Circles. Participants reported feeling disempowered and a lack of personal choice.

Even though mandates had a capacity to influence behavior, these effects did not continue for subsequent vaccine doses. A total of 52% of survey participants stated they did not intend to receive any further COVID-19 vaccine doses beyond what was mandated. Hesitancy and nuanced reactions to the mandates were also identified in the Yarning Circles. These findings suggest that while mandates were effective in increasing initial uptake, they may have also contributed to longer-term vaccine hesitancy once the COVID-19 mandate was lifted.

### 3.4 Post-pandemic challenges

Information gaps, accessibility issues related to health care, COVID-19 disease and vaccine information, and a lack of follow-up care were identified in the surveys and Yarning Circles as ongoing challenges to uptake of COVID-19 vaccinations. A key issue participants raised was about the safety of the vaccination in pregnancy and its effect on fertility.

### 3.5 Dissemination

This paper describes a study that is Aboriginal Community-led, designed, and delivered. As such, the research offers the best chance to provide biomedical knowledge in a way that is relatable, accepted, and recognized by the Community. The CAC and the HTWC guided the dissemination of findings of this research. In doing so, the study used expert knowledge to develop and implement a broad array of information and resources aimed at increasing knowledge of COVID-19 illness and increasing confidence around COVID-19 vaccinations for Aboriginal women of childbearing age, and mothers of Aboriginal children. The study results have been disseminated using a range of plain language Community reports, peer-reviewed publications relevant to Aboriginal health and methods, public health, COVID-19 vaccinations, medicine, and social science. The project's investigators and research team have also shared the research findings through organizational social media channels, mainstream media, and national and international conferences. Patient consent is not required for these research outputs, although participants have been made aware that the information produced as part of this research will be broadly disseminated.

## 4 Discussion

Aboriginal Communities face higher rates of chronic health conditions (5, 56–60), limited access to healthcare (43, 61, 62) and socioeconomic disadvantages (14, 63–65). As evidenced in the findings of this project, these factors increased vulnerability to COVID-19 during the pandemic in 2022.

The findings from the Ngarngk Koolangka Moorditj Yarning Project reveal critical insights into the factors influencing COVID-19 vaccination uptake among Aboriginal women of childbearing age. This study grounded in Aboriginal Participatory Action Research (APAR), highlights the importance of culturally relevant methodologies in addressing health disparities within Indigenous communities.

The project demonstrated that trust, Cultural Safety, Community leadership and meaningful partnerships are central to vaccine acceptance and uptake. These learnings can inform the application of Community-led and co-design approaches to routine maternal, childhood and adolescent immunization programs in Aboriginal communities, further improving vaccine confidence and accessibility beyond the COVID-19 pandemic. These findings have also informed the development of future Aboriginal-led vaccination research, reinforcing the value of Culturally safe engagement, knowledge translation and preparedness for future public health challenges.

### 4.1 The role of co-design in APAR

The methodological framework employed in this study underscores the efficacy of co-design in research. By actively involving Aboriginal women and their communities through the co-designing process, the project not only gathered valuable data but also empowered participants to shape the research. This approach not only enhances the relevance of the findings but also aligns with the principles of self-determination and Community empowerment (1, 8). The reflections from the governance body illustrate that the co-design process was instrumental in establishing trust and ensuring that the research outputs are meaningful and beneficial to the Community.

### 4.2 Methodological contributions

This study contributes to the growing body of literature advocating for Indigenous methodologies in health research. The integration of quantitative and qualitative methods via mixed-methods approaches allowed for a comprehensive understanding of the factors influencing vaccine uptake. The careful design and iterative feedback process ensured that the survey and Yarning Circle discussions remained culturally appropriate and contextually relevant. Additionally, the utilization of the NKMY framework for co-design provided a robust structure that prioritized Aboriginal voices throughout the research.

### 4.3 Implications for public health

The outcome of this research has far-reaching implications for public health initiatives targeting Aboriginal communities. The insights gained can inform the development of tailored educational resources that address specific concerns and misinformation regarding COVID-19 vaccination. It is crucial for health policymakers and practitioners to recognize the impact of culturally grounded approaches in enhancing vaccine confidence among Aboriginal women.

### 4.4 Future research directions

While the study offers valuable insights, it also highlights the need for on-going research to explore the evolving dynamics of vaccine uptake in Aboriginal populations. Future studies should consider longitudinal approaches to assess the long-term impacts of Community-led initiatives and the effectiveness of co-designed educational campaigns. Additionally, expanding the research to include a broader range of Aboriginal communities across Australia could provide a more comprehensive understanding of the diverse factors influencing vaccine acceptance.

## 5 Conclusion

In conclusion, the Ngarngk Koolangka Moorditj Yarning Project exemplifies the potential of APAR in addressing health disparities. By centering Aboriginal voices and utilizing co-design principles, this research not only contributes to academic knowledge but also serves as a blueprint for future health initiatives aimed at improving vaccination uptake and overall health outcomes for Aboriginal women and their families. The findings underscore the importance of cultural legitimacy in research and the need for sustained partnerships between Indigenous communities and health researchers to foster trust and promote health equity.

## Data Availability

The datasets presented in this article are not readily available because Data access is not available as this violates Aboriginal data sovereignty. Open data access was not included in the study information sheet at the time the survey was conducted. Request to access the data will be assessed and granted by the Cultural Advisory Committee of the research. Requests to access the datasets should be directed to A-ME, anne-marie.eades@curtin.edu.au.
