# Supplementary material for: Using Aboriginal participatory action research within a mixed methods study exploring COVID-19 vaccination uptake in Aboriginal women: a methodological framework for co-design
Source: Front Glob Womens Health. 2026 Jul 29;7:1781179. doi: 10.3389/fgwh.2026.1781179 (PMC13463408; doi:10.3389/fgwh.2026.1781179)
Supplement: Supplementary file 1 [file Supplementaryfile1.pdf]

EXPORTED ONLINE SURVEY QUESTIONS

Start of Block: Informed Consent

Q1.1

Thank you for your interest in the **Ngarngk Koolangka Moorditj Yarning** Project.

The next page will give you some important information about the project and you will be asked if you would like to take part.

Your participation is voluntary and you can stop at any time by closing the page.

Q1.2 What is the research study about?

We would like to gather information about your thoughts and experiences of the COVID-19 vaccination roll-out and how effective you think it was and what influenced your decision whether or not to get vaccinated. We would like hear from clients of South West Aboriginal Medical Service or Babbingur Mia Aboriginal Community Health program who are:

Pregnant Aboriginal women, OR breastfeeding Aboriginal women, OR Aboriginal women who have had a baby since 2019. Pregnant women from any cultural background who are pregnant with an Aboriginal child OR breastfeeding an Aboriginal child OR who have had an Aboriginal baby since 2019.

Who is conducting the research?
This research project is being led by Dr Anne-Marie Eades from the University of Technology, Sydney. The project is funded by a grant and is being run in conjunction with the South West Aboriginal Medical Service and Babbingur Mia Aboriginal Community Health program.

What will happen with what you tell us?
The information you provide will be combined with the information collected from other service providers, compiled into a report, and shared with local community members and organisations.

Research results might also be published online, talked about in the media, and/or in academic journals. No one will be able to identify you, and your information will remain confidential. No individual participant, member of staff, their organisation or individual perspective will be reported.

Standard of Care

Your standard of care will not be affected if you choose to participate in this study. Also, if you decide to withdraw from the study, the standard of care you receive from your health service will not be affected.

Further details about the study can be accessed here:

Research information sheet v2 Participant Information Form

Q1.3 By continuing with this survey, I understand:

- I am being asked to give my consent to be part of this research study.
- The purpose of the study as described in the Participants Information Form: Participant Information Form
- What the study is about, what will happen, which was described on the previous page, and in more detail by clicking the link to the Participant Information Form.

I freely agree to participate in this research study as described and understand:

- That I am free to withdraw at any time during the study by closing the survey browser.
- That withdrawal from the study will not affect my relationship or Standard of Care provided to me with any of the named organisations and /or team members.
- When the survey is completed online, it will not be possible to remove my answers once I've started as the data are anonymous.
- I can contact alison.walton-blane@uts.edu.au or speak to the community facilitator at Babbingur Mia Aboriginal Health Service or at the South West Aboriginal Medical Service to can ask questions if I need further information.
- If I would like to receive a plain language copy of the overall study results via email or post, I can contact the researchers to request this.

By continuing with this survey, I provide my consent:

- For the information collected about me to be used for the purpose of this research and that the data will be securely stored for 7 years.
- To the storage and use of my information in future ethically approved research projects related to this project.

Approval to conduct this research has been provided by the Western Australian Aboriginal Health Ethics Committee, and the University of Technology Sydney Human Research Ethics Committee in accordance with its ethics review and approval procedures.

I also understand that if I have any **concerns or complaints regarding this study,** I can confidentially contact Dr Anne- Marie Eades on 040 798 3720, anne-marie.eades@uts.edu.au or, I can contact Western Australian Aboriginal Health Ethics Committee Chair on (08) 9227 1631 or email: ethics@ahcwa.org

Yes I understand and am happy to participate
No, I am not interested

Skip To: End of Survey If By continuing with this survey, I understand: I am being asked to give my consent to be part of... = No, I am not interested

End of Block: Informed Consent

Start of Block: Demographics

Q2.1
This first set of questions will get to know a bit about you.

All the information submitted is anonymous.

____

Q2.2 What is your age (years)?

▼ 18 ... 60+

Q2.3 What do you consider to be your gender?

Female
Other (please specify)

____

Q2.4 Do you identify as an Aboriginal and / or Torres Strait Islander person?

Yes - Aboriginal
Yes - Torres Strait Islander
Yes - Aboriginal and Torres Strait Islander
No

Display This Question:

If Do you identify as an Aboriginal and / or Torres Strait Islander person? = No

Q2.5 Are you the mother of an Aboriginal child?

Yes
No

Skip To: End of Survey If Are you the mother of an Aboriginal child? = No Skip To: Q2.10 If Are you the mother of an Aboriginal child? = Yes

Q2.6 Do you identify with a ***Western Australia*** Aboriginal or Torres Strait Islander language group?

Yes
No
Not sure

If Do you identify with a Western Australia Aboriginal or Torres Strait Islander language group? = Yes

Q2.7 Which Western Australia Aboriginal or Torres Strait Islander language group(s) do you identify with? Select all that apply.

Amangu
Badimia
Bardi
Belaa

Bunuba
Gooniyandi
Juwaliny
Karajarri
Kimberly Kriol
Mangala
Martu
Wangka
Martu
Miriwoong
Naaguja
Nanda
Ngaanyatjarra
Ngaanyatjarra Pitjantjatjara Yankunytjatjara
Ngadju
Ngarinyin
Noongar / Nyoongar
Nyamal
Nyangumarta
Nyikina
Pintupi
Pitjantjatjara
Wajarri / Wadjarri
Walmajarri
Wangkatja
Yamatji
Yawuru
Yindjibarndi
Yulparija
Other (please specify) ____

Display This Question:

If Which Western Australia Aboriginal or Torres Strait Islander language group(s) do you identify wi...
= Noongar / Nyoongar

Q2.8 Do you identify with any of the following language groups? Select all the apply

Amangu
Balardong/Ballardong
Bibulmun/Piblemen
Binjareb/Pinjarup
Ganeang
Goreng
Mineng

Njunga
Nyakinyaki
Wardandi
Whadjuk/Wajuk
Wilman
Wudjari
Yued/Yuat/Juat

Q2.9 Do you identify with an Aboriginal or Torres Strait Islander language group from elsewhere in Australia?

Yes (please specify)
____ No
Not sure

Q2.10 Do you speak any languages other than English?

Yes (please specify) ____
No

Q2.11 In what region(s) of Western Australia do you normally live? (select all that apply)

Gascoyne
Goldfields - Esperance
Great Southern
Kimberley
Mid-West
Ngaanyatjarra Pitjantjatjara Yankunytjatjara (NPY) Lands
Peel
Perth Metropolitan Area
Pilbara
Southwest
Wheatbelt

Q2.12 How would you describe your day-to-day activities? (select all the apply)

Working full-time
Working part-time
Self-employed

Unemployed and looking for work
A homemaker or stay-at-home parent
Student
Retired
Other (please specify) ____

Q2.13 Which of the following best describes your household living arrangements?

Single
Single parent with children
Couple
Couple with children
Multi-generational living (multiple related adults and children)
Shared (related adults)
Shared (unrelated adults)
Other (please specify) ____

Q2.14 Do you have any medical conditions that influenced your decision whether or not to get the COVID-19 vaccination?

Yes (please specify) ____
No

End of Block: Demographics

Start of Block: Children

Q3.1 Do you have any dependent children (under 18 years)?

Yes - 1 child
Yes - more than 1 child
No

Skip To: End of Block If Do you have any dependent children (under 18 years)? = No

Display This Question:

If Do you have any dependent children (under 18 years)? = Yes - 1 child

Q3.2 Has your child had a COVID-19 infection?

Yes
No
Not sure

Display This Question:

If Do you have any dependent children (under 18 years)? = Yes - more than 1 child

Q3.3 Have your children had a COVID-19 infection?

Yes - they have all had a COVID-19 infection
Yes - some of my children have had a COVID-19 infection
No - they have not had a COVID-19 infection
Not sure

Display This Question:

If Do you have any dependent children (under 18 years)? = Yes - 1 child

And Do you have any dependent children (under 18 years)? = Yes - 1 child

Q3.4 Do you have a child/ children under 18 years eligible for the COVID-19 vaccine?

Yes
No

Skip To: End of Block If Do you have a child/ children under 18 years eligible for the COVID-19 vaccine? = No

Q3.5 Has your eligible child/ children received a COVID-19 vaccination?

Yes
No
Not sure

Display This Question:

If Has your eligible child/ children received a COVID-19 vaccination? = Yes

Q3.6 Did you child/ children experience any side effects from the COVID-19 vaccine?

Yes
No
Not sure

Display This Question:

If Do you have a child/ children under 18 years eligible for the COVID-19 vaccine? = Yes
And Has your eligible child/ children received a COVID-19 vaccination? = Yes

Q3.7 Has your child received all their doses of the COVID-19 vaccine?

Yes
No

Display This Question:

If Has your eligible child/ children received a COVID-19 vaccination? = No
Or Has your child received all their doses of the COVID-19 vaccine? = No

Q3.8 What reason has your child not had all their doses of the COVID-19 vaccine?

I don't think they should have the vaccine
I haven't had time to get the vaccine
They experienced side effects from their first dose
They got a COVID infection so they didn't need another vaccine
They have only just become eligible for the vaccine
Other (please specify) ____

Start of Block: Pregnancy and COVID-19

Q4.1 Are you currently pregnant?

Yes
No

Q4.2
Have you been pregnant since the start of the COVID-19 pandemic (i.e., any time after January 2020)?

Note: If you were pregnant but did not have the baby for any reason please select yes. You do not need to provide any further details.

Yes - I was pregnant during the COVID-19 pandemic.
No - I have not been pregnant during the COVID-19 pandemic but I was trying to get pregnant.
No - I have not been pregnant, nor have I been trying to get pregnant.

Skip To: End of Block If Have you been pregnant since the start of the COVID-19 pandemic (i.e., any time after January 202... = No - I have not been pregnant, nor have I been trying to get pregnant.

Display This Question:

If Have you been pregnant since the start of the COVID-19 pandemic (i.e., any time after January 202... = No - I have not been pregnant during the COVID-19 pandemic but I was trying to get pregnant.

Q4.3 Have you had a COVID-19 vaccine whilst trying to get pregnant?

Yes
No

Skip To: End of Block If Have you had a COVID-19 vaccine whilst trying to get pregnant? = Yes

Skip To: End of Block If Have you had a COVID-19 vaccine whilst trying to get pregnant? = No

Q4.4 Did you test positive to COVID-19 during your pregnancy?

Yes
No

Q4.5 Have you had a COVID-19 vaccine whilst pregnant?

Yes
No

Q4.6 Have you had a COVID-19 vaccine whilst breastfeeding?

Yes
No
Not applicable

Q4.7 Did you have any other vaccines whilst pregnant?

Yes (please specify) ____ No

Q4.8 How frequently have you accessed the following services during and in relation to your recent pregnancy?

	Not at all	Only Once	2-3 times	4 or more times
Aboriginal Medical Service	○	○	○	○
Allied health services (e.g., a physiotherapist, occupational therapist)	○	○	○	○
Antenatal clinic (hospital outpatient)	○	○	○	○
Community antenatal clinic	○	○	○	○

GP	○	○	○	○
Mental health service (e.g., a counsellor, psychologist, or psychiatrist)	○	○	○	○
Midwife	○	○	○	○
Pharmacy	○	○	○	○
Other - please state	○	○	○	○

Q4.9 Did the COVID-19 lock-down and related restrictions impact your ability to access pregnancy related health and medical services?

Yes (please tell us how) ____

No

Q4.10 At any time during your pregnancy, were you recommended by a health professional to get a COVID-19 vaccine?

Yes

No

Display This Question:

If At any time during your pregnancy, were you recommended by a health professional to get a COVID-1... = Yes

Q4.11 Which health professional recommended you get a COVID-19 Vaccine?

Aboriginal Health Worker

GP

Midwife

Nurse

Obstetrician

Pharmacist
Other (please specify) ____

Q4.12 Did pregnancy influence your decision whether or not to get the COVID-19 vaccine?

Yes (please tell us how) ____ No
(please tell us how) ____

Q4.13 Did the COVID-19 vaccine influence your decision whether or not to breastfeed?

Yes (please tell us how) ____ No

Q4.14 In your own words, what most influenced your decision whether or not to get a COVID-19 vaccine during your pregnancy?

____

____

____

____

____

End of Block: Pregnancy and COVID-19

Start of Block: COVID-19 Experiences

Q5.1
This next set of questions will ask you about your experiences with any COVID-19 infections and the COVID-19 vaccine.

Q5.2 Have you ever had a COVID-19 ***infection***?
Yes - once
Yes - more than once
No

Display This Question:
If Have you had a COVID-19 vaccine whilst pregnant? = No

Q5.3

Have you ever had a COVID-19 ***vaccine***?

Yes

No

Skip To: Q5.14 If Have you ever had a COVID-19 vaccine? = No

Q5.4 How many doses of the COVID-19 vaccine have you had?

One Two

Three

Four

Five

More than five

Q5.5 Which vaccine did you get? Select all that apply.

Astrazeneca

Moderna

Novavax

Pfizer

Not sure

Q5.6 Were you given the choice for which vaccine to get?

Yes

No

Q5.7 Did vaccine choice influence your decision whether or not to get vaccinated?

Yes

No

Q5.8 Where did you get your COVID-19 vaccine? (Select all that apply)

Aboriginal Health Service

GP

Mass vaccination centre
Pharmacy
Other (please state) ____ Q5.9
Did you experience any side effects related to the COVID-19 vaccine? (Select all that apply)

None
Breathlessness
Changes to menstrual cycle
Chest pain
Chills
Fever
Headache
Insomnia
Muscle pain
Nausea or vomiting
Pain at the injection site
Rash
Redness at the injection site
Swelling at the injection site
Swelling of lymph nodes in the armpit
Tiredness
Other (please specify) ____

Q5.10 Were the symptoms severe enough to report to medical professional? (select all that apply).

I reported the to West Australian Vaccine Safety Surveillance program (the survey follow up)
I sought a review by a health care provider
I did not experience symptoms severe enough to report to a medical professional Q5.11 Did you experience any of the following changes to your menstrual cycle after taking the vaccine?

Increased cycle length (late period)
Decreased cycle length (early period)
Heavier bleeding than normal
Lighter bleeding than normal
Longer bleeding than normal
Shorter bleeding than normal
Bleeding when I do not normally have a period
Other (please specify) ____ None
I do not normally have periods

Q5.12 Did you experience COVID-19 symptoms for more than 4 weeks after testing positive?
Yes
No

Q5.13 Did you experience COVID-19 symptoms for more than 3 months after testing positive?
Yes
No

Q5.14
Do you know anyone who experienced serious side effects from the COVID-19 vaccine? Yes
No

Q5.15 Do you know anyone who was seriously ill with a COVID-19 infection?

Yes
No

Q5.16 Have you had long-COVID?

Yes
No
Not sure

Q5.17 Do you know anyone who has or had long-COVID?

Yes
No

Display This Question:

If Have you ever had a COVID-19 vaccine? = No

Q5.18 What made you decide not to get a COVID-19 vaccination?

____

Display This Question:

If Have you ever had a COVID-19 vaccine? = No

Q5.19 Would anything help you chose to get a COVID-19 vaccine?

Yes (please specify) ____ No

____

End of Block: COVID-19 Experiences

Start of Block: Accessing COVID-19 Information

Q6.1
The next set of questions will ask you about what influenced your COVID-19 vaccination decision.

Q6.2
At present, there are multiple sources of information regarding COVID-19 infections and vaccines.
Did the following sources of information influence your opinion of the COVID-19 vaccine?

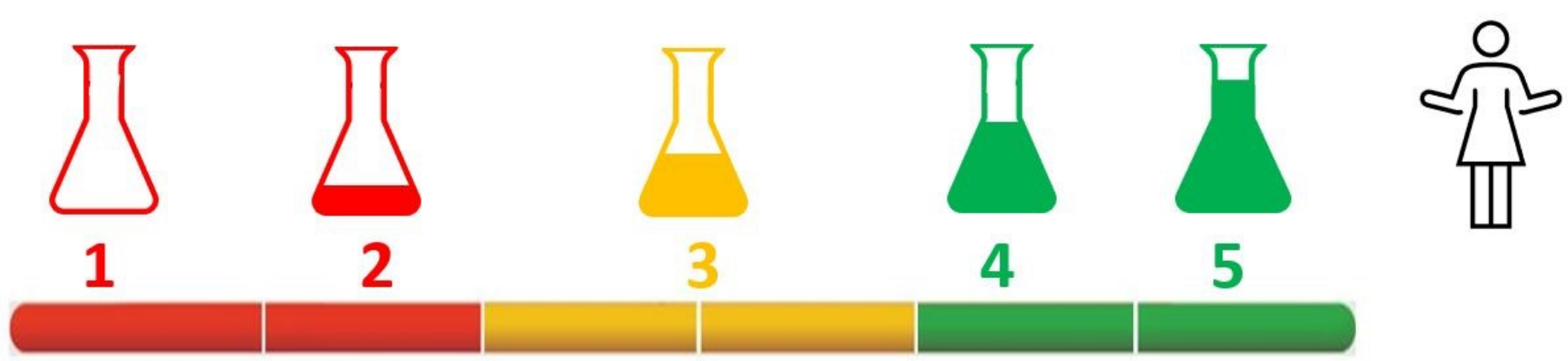

Not at all A bit A lot Not Applicable

	1	2	3	4	5	Not Applicable
Discussions with community and elders (1)						
Discussion amongst friends and family (2)						
Government agencies (3)						

Health care provider (4)						
News from National TV/Radio (5)						
News from local media providers, e.g., Noongar radio (10)						
News from Aboriginal and Torres Strait Islander specific media, e.g., NITV, Indigitube (11)						
Previous experience (6)						

Q6.3 Were you able to access information regarding the COVID-19 infection and vaccine in your preferred language?

Yes
No

Q6.4 Was the COVID-19 infection and vaccine information presented clearly in your preferred language?

Yes
No

Q6.5 Did accessing COVID-19 information in your preferred language influence your decision whether or not to get the COVID-19 vaccine?

Yes ____
No ____

End of Block: Accessing COVID-19 Information

Start of Block: Resources and Support

Q7.1 Did you receive enough information related to COVID-19 and pregnancy or conception?

Yes
No

Display This Question:

If Are you currently pregnant? = Yes

Or Have you been pregnant since the start of the COVID-19 pandemic (i.e., any time after January 202... = Yes - I was pregnant during the COVID-19 pandemic.

Q7.2 Did you receive enough support from your healthcare team whilst pregnant or trying to conceive during the pandemic?

Yes
No

Display This Question:

If Are you currently pregnant? = Yes

Or Have you been pregnant since the start of the COVID-19 pandemic (i.e., any time after January 202... = Yes - I was pregnant during the COVID-19 pandemic.

Q7.3 What additional COVID-19-related healthcare information would you have liked during your pregnancy in the pandemic?

Additional written or in-person information provided by a healthcare professional
Reminders provided for vaccination appointments Other
(please specify)

Display This Question:

If Are you currently pregnant? = Yes

Or Have you been pregnant since the start of the COVID-19 pandemic (i.e., any time after January 202... = Yes - I was pregnant during the COVID-19 pandemic.

Q7.4 What additional healthcare support would you have liked during your pregnancy in the pandemic?

Face-to-face appointments
A chosen support person during appointments
Additional mental health support
Childcare at vaccine clinics
Vaccines delivered in the home
Extended hours (evenings & weekends) to receive vaccinations
Other (please specify) ____

Q7.5 Would having additional COVID-19 related information from a healthcare professional have influenced your decision whether or not to get the COVID-19 vaccine?

Yes
No

Q7.6 Would having additional healthcare support have influenced whether or not you got the COVID-19 vaccine?

Yes
No

End of Block: Resources and Support

Start of Block: The Vaccine Mandate

Q8.1
The next set of statements are related to the COVID-19 vaccination mandate in Western Australia.
The WA COVID-19 vaccine mandate was in place from 1st December 2021 - 10th June 2022.

Please select the response that best describes your situation or level of agreement.

Q8.2
Please select the number response that best answers to the below question:

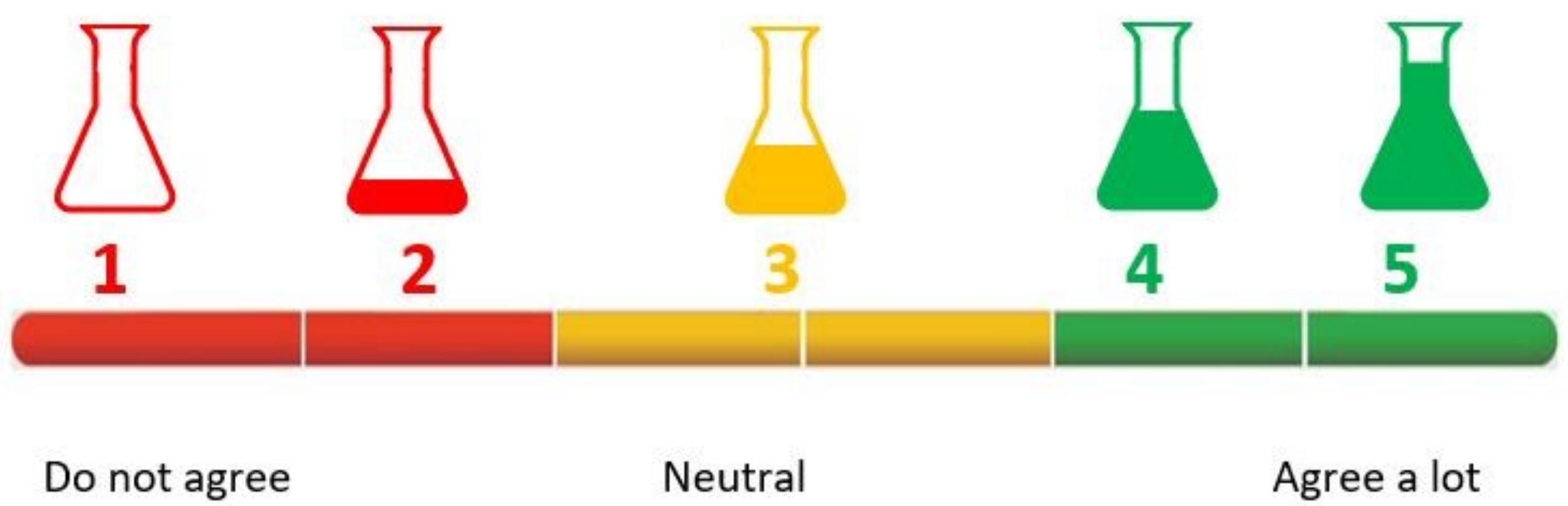

	1	2	3	4	5
The vaccination mandate in Western Australia influenced my decision to get the COVID-19 vaccine					

Q8.3
What did you do after the COVID-19 mandate was imposed?

The WA COVID-19 vaccination mandate was in place from 1st December 2021 - 10th June 2022.

- I had already taken the vaccine
- I got the vaccine to remain employed
- I refused to get the vaccine and left my employment
- Other (please specify) ____

Q8.4 Select the statement that best reflects your thoughts on the vaccine mandate.

The vaccine mandate made me more willing to get the vaccine
The vaccine mandate made me less willing to get the vaccine
The vaccine mandate did not influence my decision to get the vaccine

---

Q8.5
Do plan to get any more doses of the COVID-19 vaccine now that the Western Australian government has dropped the COVID-19 vaccination mandate?

The WA vaccination mandate was in place from 1st December 2021 - 10th June 2022. Yes
Maybe
No

Display This Question:

If Do plan to get any more doses of the COVID-19 vaccine now that the Western Australian government... = Yes

Or Do plan to get any more doses of the COVID-19 vaccine now that the Western Australian government... = Maybe

Or Do plan to get any more doses of the COVID-19 vaccine now that the Western Australian government... = No

Q8.6 Can you please explain your answer in more detail?

____

End of Block: The Vaccine Mandate

Start of Block: The Vaccination Concerns in COVID-19 Scale

Q9.1
These questions will ask for your thoughts related to various aspects of the COVID-19 vaccine.

Please select the number response that shows how much agree you with each statement:

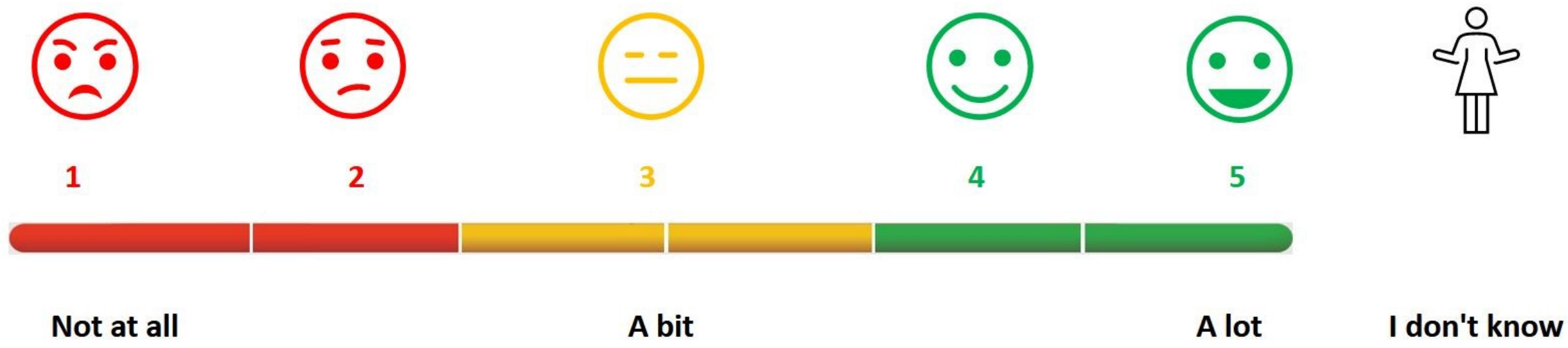

	1	2	3	4	5	I don’t know
I have access to all the information I need to make good decisions about getting the COVID-19 vaccine. (1)						
Information about the COVID-19 vaccine is easy to understand. (2)						
I don’t have enough information about the COVID-19 vaccine to decide whether to take it. (3)						

Please select the response that best reflects your level of agreement with each statement:

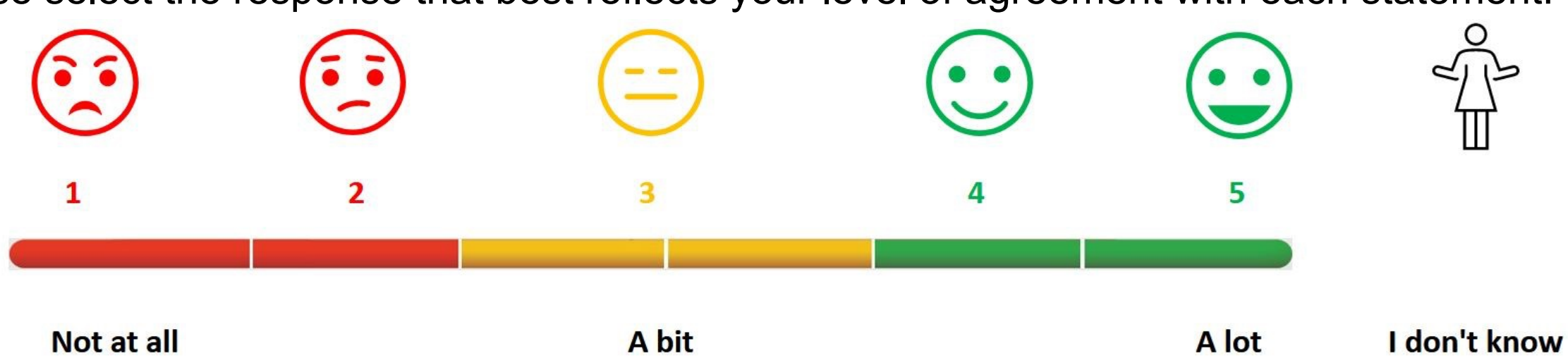

	1	2	3	4	5	I don't know
I trust the government to give me reliable information on the benefits and risks of the COVID-19 vaccine.						
I trust Healthcare Providers and Health Professionals to give me reliable information on the benefits and risks of the COVID-19 vaccine.						

I trust the government's conclusions that the COVID-19 vaccine is safe.						
I trust scientists to give me reliable information on the benefits and risks of the COVID-19 vaccine.						
I trust scientists' conclusions that the COVID-19 vaccine is safe.						
I trust Healthcare Providers' and Health Professionals' conclusions that the COVID-19 vaccine is safe						
I trust vaccine manufacturers to give me reliable information on the benefits and risks of the COVID-19 vaccine.						

Please select the response that best reflects your level of agreement with each statement:

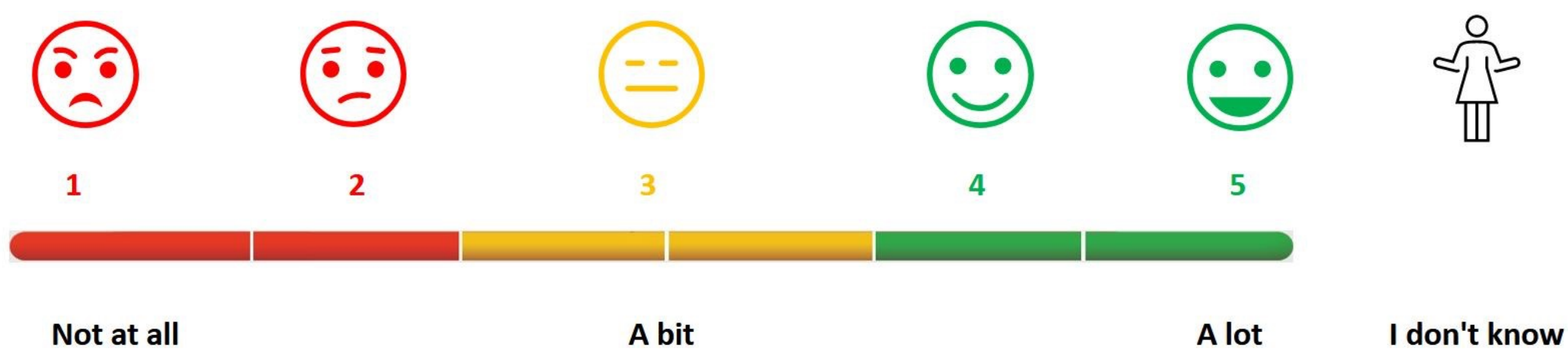

	1	2	3	4	5	I don't know
If I get the COVID-19 vaccine it will help to protect my family and friends against the coronavirus. (1)						
The COVID-19 vaccine will protect me from the coronavirus. (2)						
The COVID-19 vaccine will stop the spread of the coronavirus. (3)						
The COVID-19 vaccine is effective. (4)						

The COVID-19 vaccine will reduce the severity of symptoms if I get the coronavirus. (5)						
Getting the COVID-19 vaccine will help to get things back to normal. (6)						
It is important to get the COVID-19 vaccine so that outbreaks do not occur. (7)						
Getting the COVID-19 vaccine is important for the health of others in my community. (8)						

Please select the response that best reflects your level of agreement with each statement:

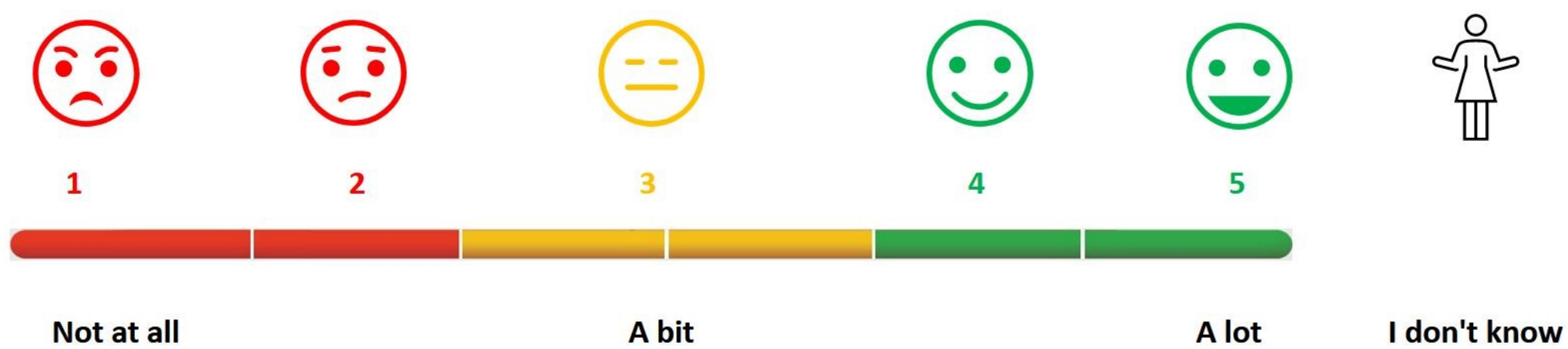

	1	2	3	4	5	I don’t know
I am concerned about the side effects of the COVID-19 vaccine. (1)						
I fear that the COVID-19 vaccine will cause side effects. (2)						
I am worried about the safety of the COVID-19 vaccine. (3)						
I am afraid of getting the vaccine (4)						
Getting the vaccine makes me anxious (5)						

Please select the response that best reflects your level of agreement with each statement:

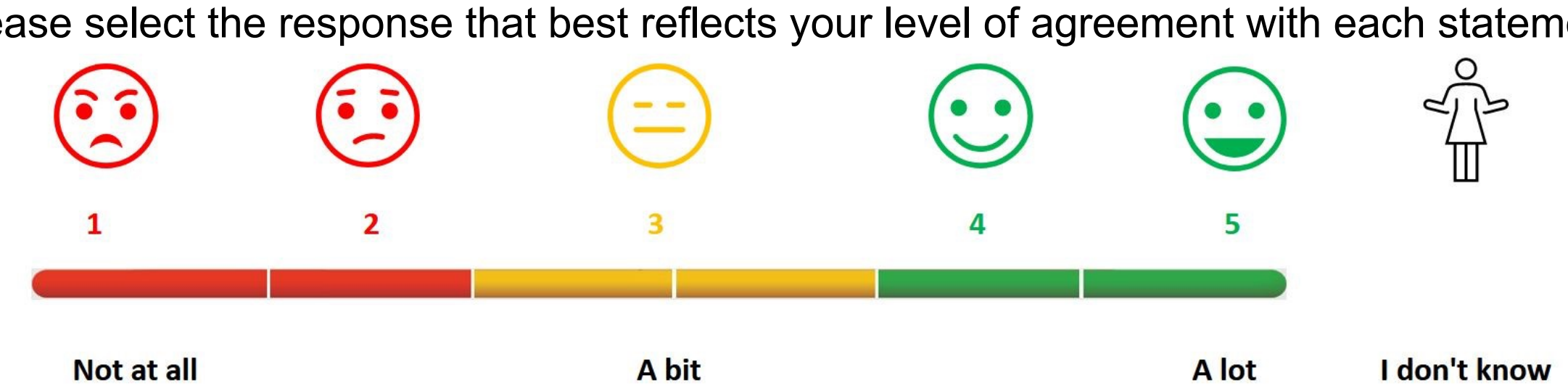

	1	2	3	4	5	I don’t know

The COVID-19 vaccine can cause the coronavirus in some people. (1)						
The COVID-19 vaccine can give you a serious case of the very same virus you're trying to avoid (2)						
I can get the coronavirus from the COVID-19 vaccine. (3)						
The COVID-19 vaccine is too new so I should wait before deciding to get it. (1)						
More time is needed to be able to fully investigate the true effects of the COVID-19 vaccine. (2)						
I am afraid that the COVID-19 vaccine has not been successfully tested on enough people. (3)						

Please select the response that best reflects your level of agreement with each statement:

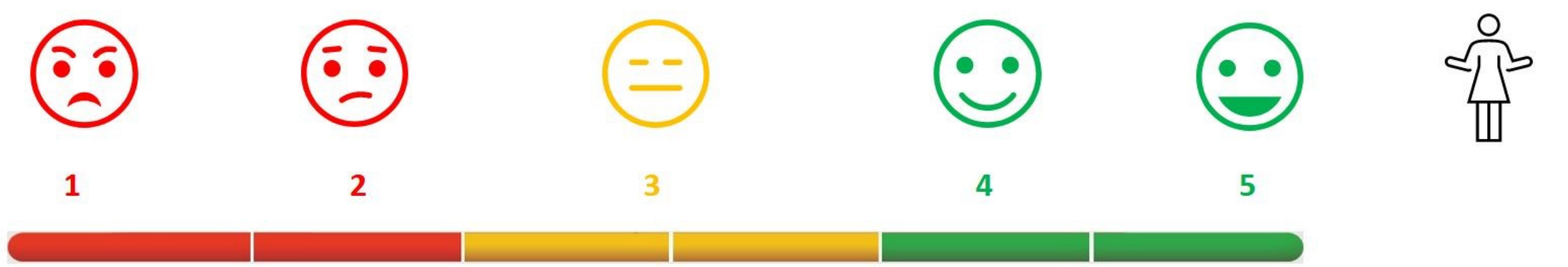

Not at all **A bit** **A lot** **I don't know**

	1	2	3	4	5	I don't know
The COVID-19 vaccine safety data is often made up. (1)						
People have been deceived about the safety of the COVID-19 vaccine. (2)						
The COVID-19 vaccine is promoted mainly because of manufacturers' profit. (3)						
The main reason for promoting the COVID-19 vaccine is for drug companies to make money (4)						
I am opposed to the COVID-19 vaccine because it goes against freedom of choice. (5)						

End of Block: The Vaccination Concerns in COVID-19 Scale

Start of Block: Anything else?

Q10.1 Is there anything else about your experience with COVID-19 infections or the vaccine during pregnancy or conception that you would like to tell us?

____

End of Block: Anything else?

Start of Block: Safety Check

Q11.1 Have you completed this survey whilst at an Aboriginal Health Service?

Yes
No

End of Block: Safety Check

Start of Block: Debrief Form

Q12.1
This section is just to check on your health and wellbeing after completing this survey. Please answer the questions and let us know if you need any additional support.

Q12.2 After completing the survey, are you feeling OK?

Yes
No

Q12.3 After completing the survey, do you feel upset or distressed at all?

Yes
No

Q12.4 After completing the survey, do you feel concerned about your social and emotional wellbeing?

Yes
No

Q12.5 After completing the survey, would you like to talk to a counsellor?

Yes
No

Display This Question:

If After completing the survey, would you like to talk to a counsellor? = Yes

Q12.6 Do you consent to a counsellor referral? If yes, please let us know your organisation preference.

Yes ____ No

Q12.7 To make sure you are OK, do you want us to contact you tomorrow? If yes, what is your name and contact telephone number?

Yes ____ No

End of Block: Debrief Form
